# Pressure enabled drug delivery (PEDD) of nelitolimod increased therapeutic delivery, reduced immunosuppression, and improved efficacy in porcine and murine liver tumor models

**DOI:** 10.3389/fonc.2025.1655794

**Published:** 2025-10-06

**Authors:** Lauren Cournoyer, Yujia Liu, David B. Jaroch, Tom G. Hullinger, Bryan F. Cox, Steven C. Katz, Jason LaPorte, Ilan B. Layman, Alizee Ballarin, Chandra C. Ghosh, Prajna Guha

**Affiliations:** ^1^ Department of Surgery, Brown University School of Medicine, Providence, RI, United States; ^2^ TriSalus™ Life Sciences, Inc., Westminster, CO, United States

**Keywords:** loco-regional therapy, NIR-tissue imaging, oncopig, liver metastases, tumor microenvironment, SD-101, nelitolimod, TLR9 agonist

## Abstract

**Introduction:**

Direct tumoral needle injection of nelitolimod, also known as SD-101, has been evaluated in patients with superficial malignancies, including cutaneous melanoma, with encouraging outcomes. Direct intra-tumoral injections may not be suitable for primary and metastatic liver tumors due to the number and size of the lesions, and the location of target immune cells in the peri-tumoral parenchyma. Conventional systemic infusion may be inadequate due to high intra-tumoral pressure and distribution of the therapeutic in large quantities to non-target tissue. Previous clinical and preclinical reports suggest that nelitolimod favorably reprograms the tumor microenvironment to limit myeloid-induced immunosuppression and promote anti-tumor immunity. The present study was undertaken to further explore the contribution of pressure-enabled drug delivery (PEDD) to the immune and clinical effects of nelitolimod in intrahepatic malignancy.

**Methods:**

Transgenic pigs (oncopigs) with liver tumors received intra-arterial infusions of fluorescently labeled ODN2395 or nelitolimod either via PEDD with a specialized infusion device or with conventional microcatheter delivery in both lobar and selective infusions. Near-infrared imaging assessed tissue distribution. The murine liver metastasis (LM) model was developed by injecting MC38-Luc cells into the C57/BL6 spleen and treating with fluorescently labeled nelitolimod (30µg/mouse). Tumor burden was monitored by an *in vivo* imaging system, serum cytokine levels were analyzed by Luminex, and blood chemistry was measured. Liver CD45+ cells were analyzed by flow cytometry to evaluate the tumor microenvironment.

**Results:**

Our results demonstrate that PEDD enhanced the intravascular infusion of nelitolimod into the liver tumor and peri-tumoral tissue. PEDD resulted in a significant increase in distribution and signal intensity (a surrogate for concentration) in target tissue compared to needle injection or a standard catheter in the oncopig model. PEDD was also modeled in the murine setting with a pressure-controlled infusion system. Single treatment of nelitolimod via PEDD, significantly reduced tumor progression as compared to systemic administration. PEDD of nelitolimod significantly reduced immunosuppressive MDSCs and an increase in cytotoxic CD8 + T cells within the LM. In conclusion, use of PEDD enhanced targeted therapeutic delivery in swine liver tumors and reduced tumor progression by promoting anti-tumor immunity in murine LM in association with suppressive myeloid cell elimination.

## Introduction

Synthetic class C TLR9 agonists, such as nelitolimod and ODN2395, have similar functions and activate innate and adaptive immune cells, resulting in canonical effects such as stimulating cytokine production and potentiating anti-tumor efficacy (1). Nelitolimod is a synthetic 30-nucleotide phosphorothioate oligodeoxynucleotide (ODN) containing multiple cytidine-phospho-guanosine (CpG) motifs with flanking nucleotides in a self-complimentary (palindromic) sequence. Nelitolimod is a class C agonist (TLR9C), that activates dendritic cells and B cells while reducing suppressive myeloid cell populations (2–5). As a consequence, anti-tumor CD8^+^ T cell function is enhanced in association with the elimination of myeloid-derived suppressor cell (MDSC) populations (6, 7). Nelitolimod has undergone multiple investigations for use in the treatment of solid tumors in both animal models and human clinical trials (8, 9). In these investigations, solutions carrying the active nelitolimod oligo were injected directly into the tumor mass. Due to this mode of delivery, most investigations were limited to large, easily discernable tumors located close to the external surface of the body. Such methodology is considered impractical for the treatment of multifocal visceral tumors as often found in patients with LM.

Immune checkpoint inhibitors (CPIs) have revolutionized treatment for many types of cancer but their efficacy in liver tumors has been limited. Solid tumors in the liver present several challenges for the delivery of immunomodulatory agents. The liver is an immunologically suppressed organ with myeloid derived suppressor cells (MDSCs) dampening recruitment and activation of T and NK cells in the setting of malignancy while promoting the production of regulatory T cells, all of which contribute to an immunologically cold microenvironment that likely inhibit the efficacy of CPI. In addition to the unfavorable immune environment, liver tumors are generally not amenable to direct injection due to the number and size of lesions. Also, solid tumors like those in the liver are not practical to treat by direct injection and have increased intertumoral pressures that may inhibit tumor penetration of therapeutics with intravascular delivery approaches, either systemic IV or local regional intra-arterial delivery with conventional endhole catheters (10, 11).

In order to improve immunotherapy drug responses in liver tumors (both the immunosuppressive environment and the drug delivery challenges of targeting and overcoming elevated intertumoral pressure), TriSalus Life Sciences has developed infusion technologies that modulate intravascular pressure within the target infusion zone. PEDD is achieved through using the TriNav Infusion system, which is equipped with a SmartValve^®^—a one-way dynamic microvalve that modulates intravascular pressure and flow to overcome the barrier of elevated intra-tumoral pressure. The SmartValve’s design creates turbulent flow and reopens collapsed tumor micro-vessels, enabling deeper and more uniform therapeutic penetration into tumor and peri-tumoral tissue without reflux into non-target areas. This mechanism has been demonstrated to enhance the tumor-to-normal (T:N) delivery ratio and improve uptake of microspheres and large biologics in preclinical and clinical models, thereby providing a mechanistic rationale for the improved intravascular infusion of nelitolimod described herein (12). PEDD devices have been shown in small prospective and retrospective clinical studies to reduce therapeutic delivery to nontarget normal tissues while increasing the concentration and penetration of therapeutics into the tumor vascular tree (13, 14). Phase 1 trials for primary and metastatic liver tumors and pancreatic tumors were opened to explore the use of PEDD with intravascular nelitolimod delivery (15–17).

Direct hepatic arterial infusion of therapeutic agents has been investigated with mixed success in the absence of PEDD (18). While such methodology is able to target the vasculature feeding tumor tissues, it cannot sufficiently address physical barriers to drug delivery such as high interstitial fluid pressure and solid stress which results in vascular collapse (11, 19). The use of a PEDD device for hepatic arterial infusion of nelitolimod offers a means of treating diffuse disease within the liver tissue while overcoming the barriers to drug delivery found in solid tumors (20). To evaluate the impact of a PEDD device on therapeutic distribution, a porcine model was selected based on similarities in liver vasculature, cellular structure, and internal organ structure relative to the human liver (21). Porcine models have been used extensively in investigations of local delivery of therapeutics (22–24). The liver vascular anatomy is compatible with the size range indication for the PEDD TriNav device (1.5-3.5mm diameter). Thus, effective delivery of nelitolimod via PEDD is expected to improve therapeutic delivery and promote beneficial alterations in the tumor microenvironment. Moreover, for TLR9 agonists to be successfully implemented in patients with liver tumors, immune cells within the tumor and peritumoral tissue should be targeted.

The studies were designed to test the following hypotheses: 1) PEDD via hepatic artery infusion of a TLR9C agonist provides a viable alternative to direct needle injection in liver tissue, 2) PEDD using a TriNav device via hepatic artery infusion of nelitolimod would improve uptake of nelitolimod in liver tumors and peri-tumoral parenchyma when compared to delivery with a conventional traditional microcatheter (TMC) and 3) PEDD of nelitolimod would enhance immune effects and tumor control in a murine model of LM.

To test our hypothesis, we used both porcine and murine liver cancer models. We used the PEDD Oncopig Cancer Model (OCM) to evaluate the PEDD mediated administration of nelitolimod locoregionally to the liver tumor.

The PEDD technique significantly enhanced the delivery of nelitolimod in porcine tissue and controlled tumor progression by inhibiting the immunosuppressive TME in mice. Therefore, PEDD mediated delivery of nelitolimod as monotherapy or in conjunction with neoadjuvant and adjuvant therapies has the potential to slow the progression of LM.

## Materials and methods

### PEDD vs. direct needle injection of TLR9C agonist in porcine liver

This study was performed on 8–11-week-old female Yorkshire Cross swine ranging from 45–65 kg (Oak Hill Genetics, Ewing, IL). The acute procedures described in this study have been approved by the Inotiv IACUC as part of protocol #1950. Under anesthesia, the TLR9C agonist ODN2395 oligonucleotide fluorescently labeled with IRD800 or nelitolimod labeled with Cy5.5 (Integrated DNA Technologies, Coralville, IA) was injected directly with a needle (2.5 nmol in 3 ml saline) under ultrasound guidance into the liver. For PEDD infusion, a femoral approach was used to access the hepatic vasculature under fluoroscopic guidance. A TriNav device was placed in the target hepatic artery branch, and 2.5 nmol of TLR9 agonist in 10 ml saline was infused at 2 ml/min. 90 min post-infusion, the liver was collected, and 1 cm thick sequential liver sections underwent near IR imaging with the Pearl Trilogy Imaging System (Li-Cor, Inc. Lincoln, NE) for analysis of fluorescence signal intensity and coverage area. A custom-designed MatLab (Math-Works, Natick, MA) graphical user interface (GUI) was developed to identify these discrete regions of signal. An intensity threshold was determined using an untreated tissue reference to identify the presence of the oligo fluorescence signal.

Signal arising from the treated target tissue was established for the 700nm (Cy5.5-nelitolimod) and 800 nm (IRD800-ODN2395) tool compounds. The total signal intensity in luminous units (lu) was calculated by the summation of the lu for pixels meeting threshold criteria. This produced data correlated to the signal observed for the needle injection (n=4 for IRD800-ODN2395, n=4 for Cy5.5-nelitolimod, n=8 total measurements) and the HA infusion (n=4 for IRD800-ODN2395, n=4 for Cy5.5- nelitolimod, n=8 total measurements). The mean lu for the two sides of each slice was determined. The summation of these measurements for all slices was then calculated for each subject, and the lu totals for the two infusion methods were then compared using a two-sample equivalence test at a 95% confidence interval.

The volume of coverage was calculated by taking the mean of the number of pixels displaying intensity greater than the threshold value for the two sides of each slice. This value was then converted into a tissue volume (85 µm x 85 µm pixel X 1 cm slice depth) for all tissue slices needle injection (n=4 for IRD800- ODN2395, n=4 for Cy5.5- nelitolimod, n=8 total measurements) and the HA infusion (n=4 for IRD800-ODN2395, n=4 for Cy5.5- nelitolimod, n=8 total measurements). Tissue volume for the two infusion methods was then compared using a two-sample equivalence test at a 95% confidence interval.

### PEDD with TriNav vs. conventional delivery of nelitolimod to liver tumors in the oncopig

The study was conducted in male and female transgenic pigs (oncopigs) 40–70 kg in weight. The oncopig is a transgenic model with Cre recombinase inducible KRASG12D and TP53R167H mutant oncogenes. Hepatic tumors were induced using the methodology presented by Nurili et al. (25), with transformed biopsies being injected percutaneously under ultrasound guidance back into the liver at 4 locations to induce tumor formation. 8–10 days post tumor induction, animals were anesthetized, and femoral access was used to place the delivery device in the target hepatic artery branch. 2.5 nmol of IRD800CW labeled nelitolimod + 4 mg unlabeled nelitolimod in 10 ml of saline was infused at 2 m/min using a TMC or via PEDD using a TriNav device. In the TriNav group, a rapid 1 cc infusion of saline after the first 4 ml of infusion was used. A slow saline flush was used at the end of the 10 ml infusion in both the TriNav and TMC groups. One hour post-infusion, liver tissue was collected, and serial 1 cm thick liver sections were cut. The slice of tissue containing the largest diameter cross-section of the target tumor was identified. The 800 nm channel image from the Pearl imaging system and a corresponding full color image of the slice face was identified and processed through a custom ImageJ program to produce images readable by Visopharm software (Visiopharm Co. Hoersholm, Denmark). The Visopharm Deep Learning algorithm was used to identify the tumor border, and 1 mm concentric zones extending into and away from the tumor were delineated for data processing of surface area and near-IR signal intensity.

### Effect of PEDD of nelitolimod vs. tail vein delivery in a murine model

C57BL/6J (stock number: 000664), aged 8–12 weeks, were purchased from Jackson Laboratories (Bar Harbor, Maine, USA) and housed under pathogen-free conditions in the Association for Assessment and Accreditation of Laboratory Animal Care (AAALAC)-approved Animal Research Facility at Lifespan, Coro East (Rhode Island, USA). All surgical procedures were performed as per Lifespan’s Institutional Animal Use and Care Committee’s approved protocol (Protocol#: 5010–22).To develop a murine liver metastasis model, 1e6 MC38 cells were delivered via splenic injection followed by splenectomy as performed earlier (6, 26). These syngeneic colorectal cells express luciferase to monitor tumor progression with IVIS Spectrum CT. Briefly, tumor luminescence was detected by injecting D-luciferin (150 mg kg−1, PerkinElmer, CT, USA) intraperitoneally and captured with the IVIS Spectrum CT system after 10 min. Seven days after tumor cell inoculation, 30 µg of fluorescently labeled nelitolimod was delivered to liver metastasis (LM) bearing mice either via portal vein (PV) or systemically via tail vein (TV). Blood was collected on D3, i.e. three days after PEDD delivery of labeled nelitolimod via the PV. Mice were sacrificed on D14 (fourteen days after PV infusion).

### Antibodies for flow cytometry in the murine model

Antibodies used to determine the following antigens, along with clones, are given below.

Gr1 (Ly6G/Ly6C, RB6-8C5), CD11b (M1/170), and CD3 (17A2) were obtained from BD Biosciences (CA, USA). Antibodies against CD8 (53-6.7), CD4 (GK1.5), and Zombie NIR (viability dye) were procured from Biolegend, CA, USA. At D14, blood and liver were collected. Non-parenchymal cells (NPCs) were isolated from the liver, followed by isolating CD45^+^ cells using immuno-magnetic beads (Miltenyi Biotech, MA, USA) as described earlier (27). Flow cytometry (Cytoflex, Beckman Coulter, IN, USA) was performed to determine MDSCs, CD8^+^ and CD4^+^ T cells.

### Blood chemistry and cytokine measurement in the murine model

Blood chemistry for ALP, AST, ALT, Creatine kinase, Albumin, Total Bilirubin, Total Protein, Globulin, BUN, and Creatinine was measured by IDEXX (Grafton, MA, USA). Circulatory cytokines such as IFNγ, CXCL-10 (IP-10), IL-6, IL12, IL-18, IL-2R, and MCP1 were analyzed using Procartaplex Luminex kit (Thermo Fisher Scientific, MA, USA) and were measured by Magpix (Luminex Corp, TX, USA).

### Statistics

Statistical analyses were conducted using Minitab (Minitab LLC, Chicago, IL) and Prism (GraphPad, San Diego, CA) software. One-tailed paired t-tests (consistent with the hypothesis of increased delivery) between regions of interest in the end hole and TriNav sample groups for the oncopig studies were used. For the murine studies, multiple unpaired t-test, two-stage step-up procedure of Benjamini, Krieger, and Yekutieli, with Q = 1% were performed. Prism (V.10) software (GraphPad, San Diego, California, USA) was used to analyze data. For all studies, values of p<0.05 were considered statistically significant.

## Results

### PEDD using TriNav improved the distribution of TLR9 C agonists compared to systemic delivery (needle injection) in swine liver

**Figure 1:**
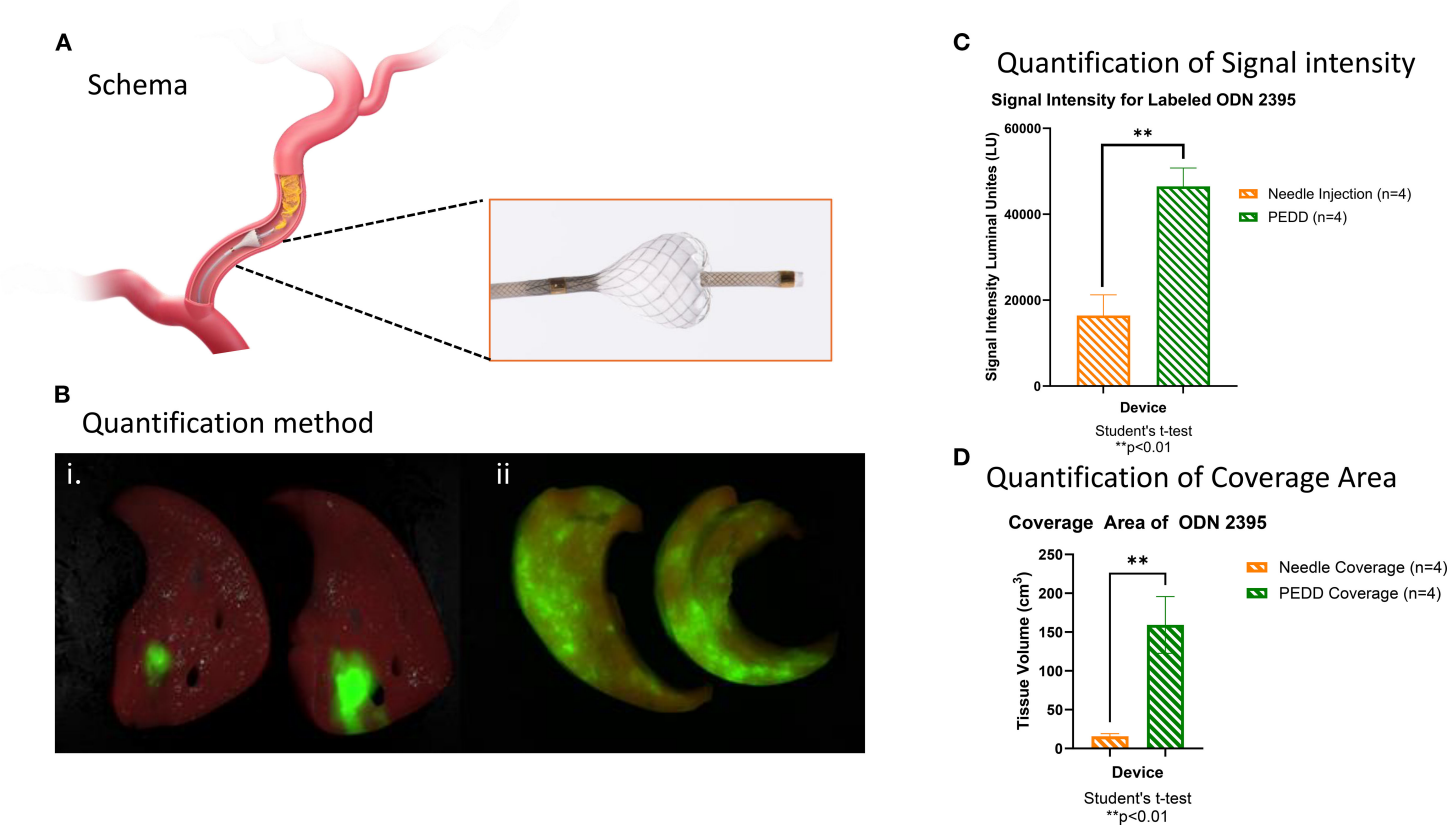
PEDD using TriNav improved the distribution of TLR9 C agonists compared to systemic delivery (needle injection) in swine liver. **(A)** Schematic representation of the TriNav Infusion System with Smart Valve. **(B)** Representative images of labeled TLR9 agonist ODN2395 delivered to the porcine liver via needle injection (i) vs. PEDD (ii). **(C, D)** Quantification of treatment delivery signal intensity and coverage area using needle injection vs. PEDD.

As shown in **Figure 1A**, the TriNav infusion system was placed in the hepatic artery, and 2.5 nM of labeled ODN2395 was delivered. As presented in **Figure 1B**, PEDD using the TriNav device appeared to increase the amount of tissue treated with ODN2395. PEDD resulted in statistically significant increases in both signal intensity (46483 ± 4285 lu vs.16438 ± 4793 lu; p<0.01) (**Figure 1C**) and tissue coverage measured for labeled ODN2395 (159.2 ± 36.6 cm^3^ vs. 15.7 ± 3.3 cm^3^; p< 0.05) when compared to needle injection (**Figure 1D**).

### PEDD with TriNav was superior to conventional microcatheter (endhole) for intra-arterial delivery of nelitolimod in oncopig liver tumors

**Figure 2:**
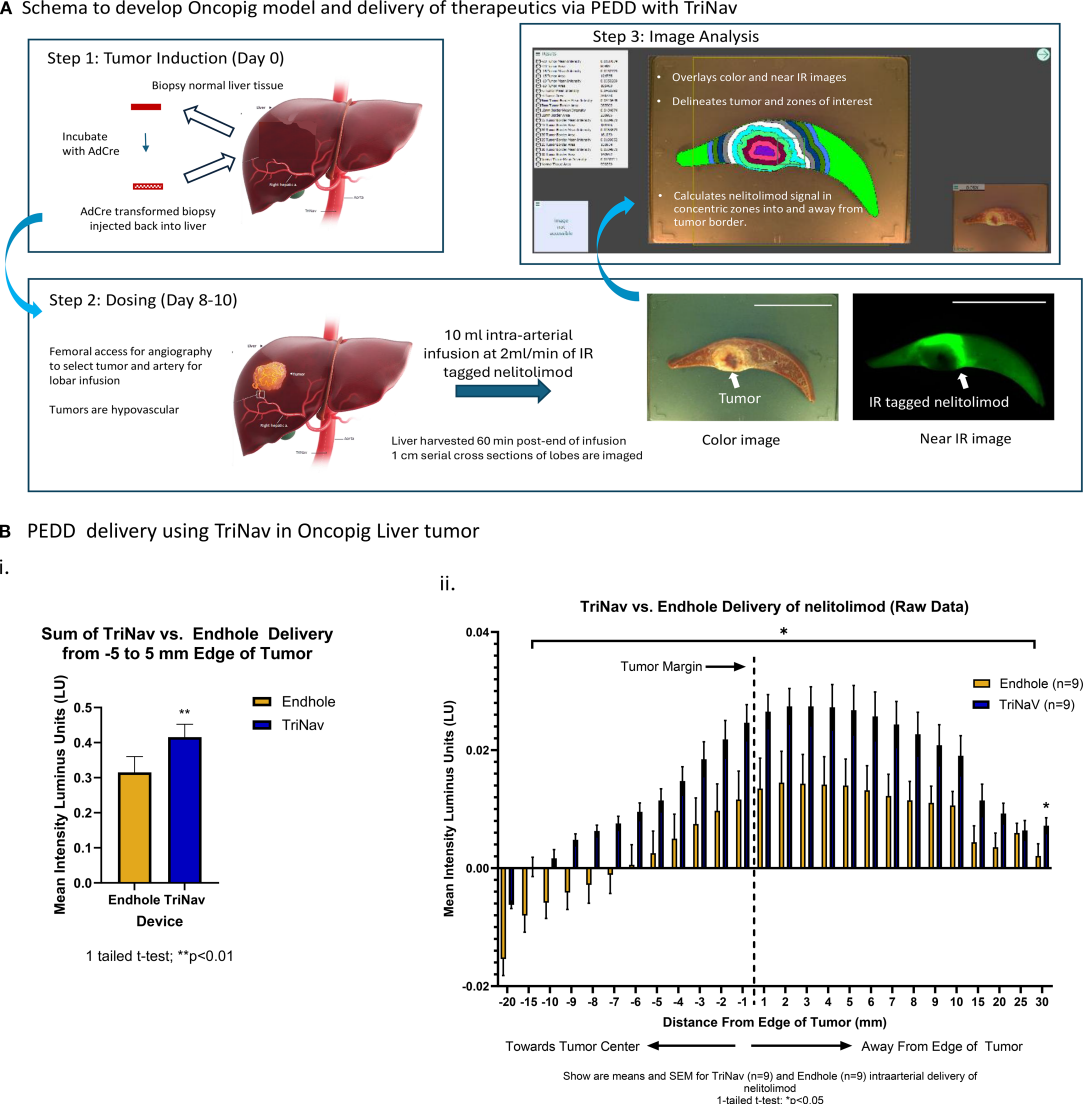
PEDD with TriNav was superior to endhole catheter for intra-arterial regional delivery of therapeutics in oncopig liver tumors. **(A)** Schematic illustration of the oncopig model development and data processing methodology. Tumors are induced in the transgenic oncopig by collecting liver biopsies, which are re-injected into the liver after incubation with AdCre. Treatments are administered after 8–10 days of tumor growth. One-centimeter cross-sections of the liver tissue are imaged in bright field to obtain high-resolution tumor outlines using the algorithm, which were used to quantify treatment signals detected in near-IR images within corresponding 1mm zones extending from the tumor margin. Scale (5 cm). **(B)** Quantification of labeled nelitolimod delivery via PEDD using the TriNav Infusion System compared to a traditional microcatheter. i. Total signal intensity summed across the -5 to 5 mm zone measured from the tumor margin. ii. Signal intensity measured in 1 mm zones from the tumor margin. The vertical dash line at 0 on the X axis represents the tumor margin, each pair of the two bars represents signal intensity measured in the 1mm band zone away from the tumor margin/previous 1mm zone. Data are presented as mean ± SEM for TriNav (n=9) and TMC (n=9) intra-arterial delivery of labeled nelitolimod (* indicates *p* < 0.05).

We compared the delivery and distribution of labeled nelitolimod via TriNav mediated PEDD in a liver tumor model developed in oncopig, as illustrated in **Figure 2A**. The mean signal intensities from 5 mm into the tumor to 5 mm away from the outer edge were significantly greater in the TriNav dosed group when compared to the conventional microcatheter dosed group (0.415 ± 0.37 lu vs. 0.315 ± 0.045 lu; p ≤ 0.01) as shown in **Figure 2Bi**. To compare the distribution of nelitolimod delivered via TriNav or TMC over normal tissue, we normalized to the mean fluorescent signal in normal tissue that was greater than 30 mm away from the edge of the tumor. We found that PEDD resulted in a statistically significant increase in nelitolimod delivery from 15 mm into the tumor to 20 mm away and 30 mm away from the outer edge of the tumor **Figure 2Bii** (p ≤ 0.05). This distribution pattern reflects the ability of PEDD to transiently overcome elevated intratumoral pressure, reopen collapsed microvessels, and promote outward diffusion of therapeutics into peri-tumoral regions. This data demonstrated that TriNav delivery of nelitolimod to liver tumors in the oncopig via PEDD is superior to a TMC.

As shown in **Figure 2B**, the sum of the mean signal intensities from -5 mm into the tumor to 5 mm away from the outer edge of the tumor were significantly greater in the TriNav dosed group when compared to the TMC dosed group (p ≤ 0.05). To further assess the delivery of nelitolimod to tumor tissue vs. normal liver tissue, the data was normalized to the mean fluorescent signal in normal tissue that was greater than 30mm away from the edge of the tumor. When the mean intensity of the nelitolimod signal was normalized to a remote normal tissue signal, PEDD resulted in statistically significant increases in nelitolimod delivery from -15 mm into the tumor to 20 mm away and 30 mm away from the outer edge of the tumor (**Figure 2Bii**, p ≤ 0.05). The sum of the mean nelitolimod signal intensity from 20 mm into the tumor to 30 mm away from the tumor with PEDD was also significantly increased when compared to the TMC delivery (**Figure 2Bii**, p ≤ 0.01).

### PEDD nelitolimod was superior to systemic nelitolimod at inhibiting tumor growth

**Figure 3:**
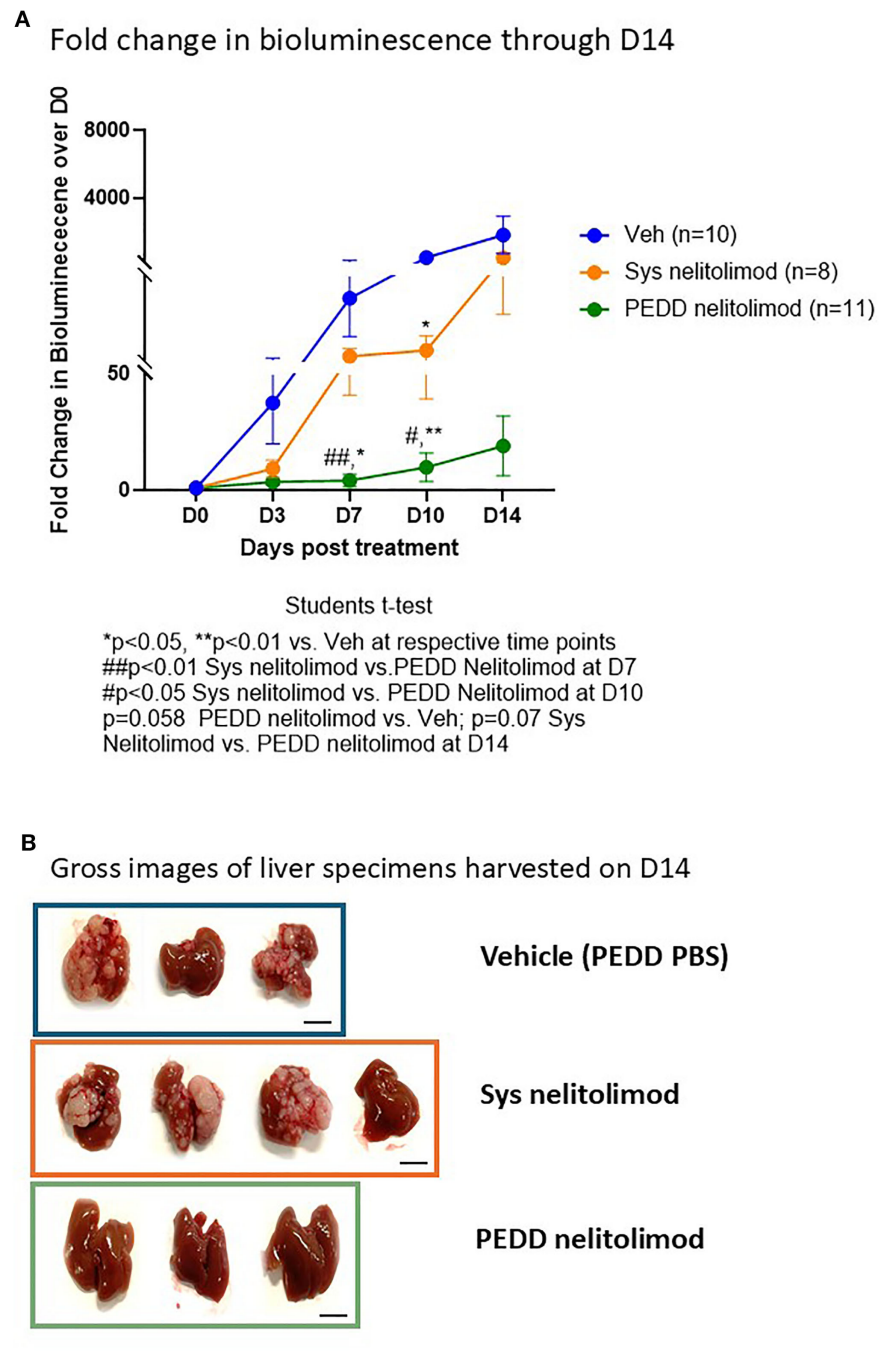
PEDD nelitolimod controlled the tumor progression better than systemic nelitolimod. **(A)** Bioluminescence values were determined by IVIS on D0, D3, D7, D10 and D14. PBS delivered via PV using PEDD served as Veh control. Fold change of the tumor burden was calculated based on D0 baseline bioluminescence (total flux/sec). **(B)** Mice were sacrificed on D14, and representative images (n=3–4 per group) depicting the gross images of the harvested livers. Scale (1 cm). Data presented as mean ± SEM; and p value is mentioned in the graph.

Mice were treated on D0 with either vehicle or fluorescently tagged nelitolimod by tail vein or with PEDD via the portal vein. Tumor progression was assessed on Days 0, 3, 7, 10 and 14. As shown in **Figure 3A**, PEDD of nelitolimod significantly reduced tumor growth on Day 7 (p<0.01) and Day 10 (p<0.05) vs. systemic nelitolimod. We found that on D10 nelitolimod, irrespective of the delivery method, significantly controlled tumor progression compared to Veh (**Figure 3A**). However, at D14 (14 days after nelitolimod treatment), systemic nelitolimod failed to control tumor progression, as demonstrated by bioluminescence images (**Supplementary Figure 1**) and gross images at D14 (**Figure 3B**).

### Nelitolimod triggered TLR9 activation

**Figure 4:**
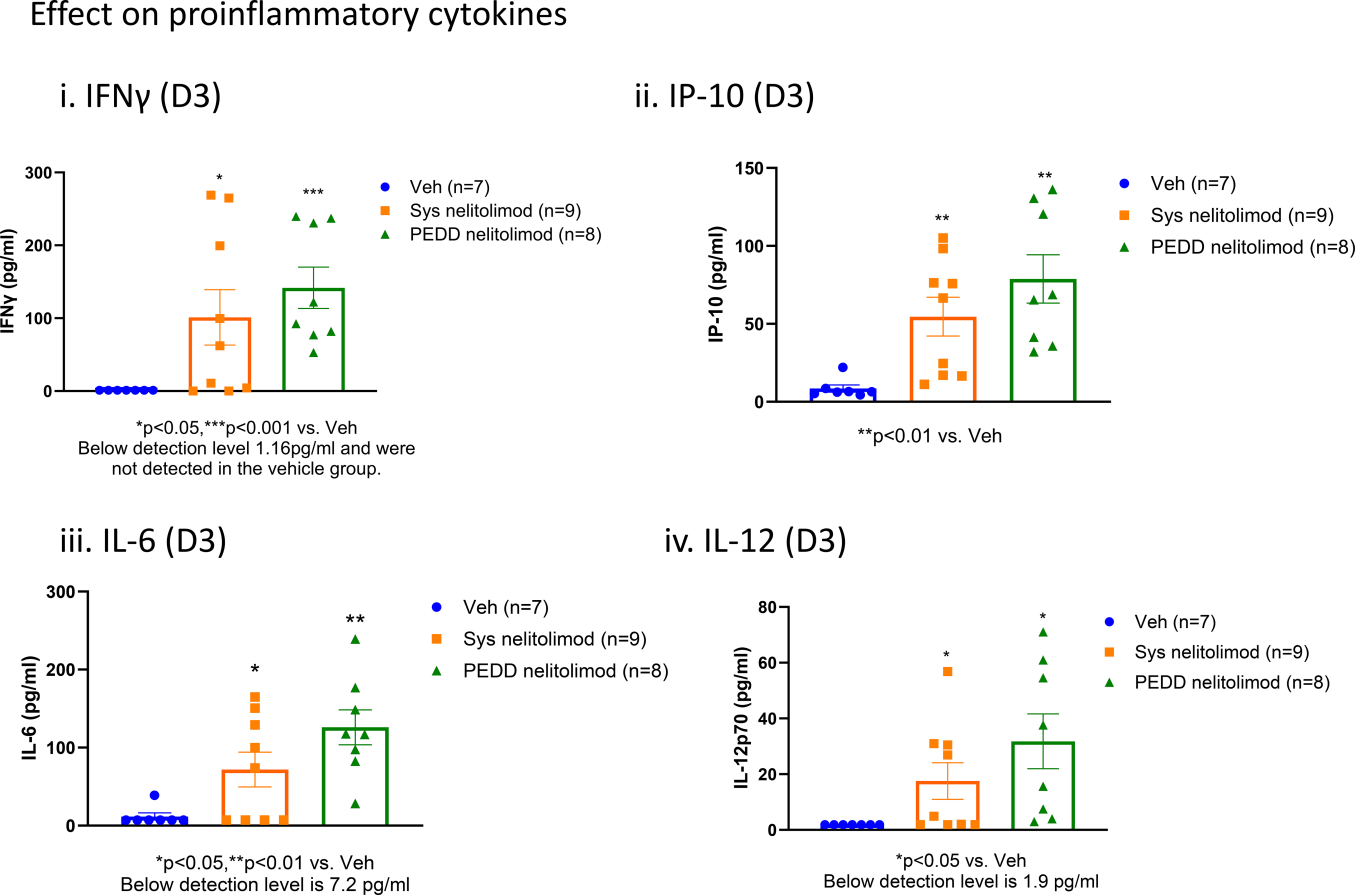
Peripheral effects of nelitolimod delivered by PEDD. Mouse serum collected at D3 was analyzed by Luminex for circulatory IFNγ (i), IP-10 (ii), IL-6 (iii), IL-12 (iv), and IL18 (v). Data presented as mean ± SEM; and p value is mentioned in the graph.

As shown in **Figure 4i-iv**, nelitolimod, irrespective of the route of delivery, increased circulatory levels of pro-inflammatory cytokines such as IFNγ, IP-10, IL6, and IL12 at D3 when compared to systemic delivery of vehicle suggesting both routes of administration were likely engaging the TLR9 receptor and triggering pro-inflammatory signaling pathways. At D14, no differences in pro-inflammatory cytokines were observed in the nelitolimod treated groups vs. the vehicle treated group (data not shown). Nelitolimod, regardless of route of delivery, did not affect the metabolic makers bilirubin, total protein, albumin, globulin, BUN, the liver enzyme ALT or creatine kinase Nelitolimod modestly but significantly increased AST at D3 vs. vehicle treatment (Veh (n=7): 128.7 ± 43.1 U/L; nelitolimod Sys (n=7): 847 ± 269.7 U/L (n=9); nelitolimod PEDD (n=9): 349 ± 76.4 U/L) but returned to normal by D14 (Veh (n=7): 419.6 ± 95.5 U/L; nelitolimod Sys (n=7): 261.6 ± 46 U/L (n=9); nelitolimod PEDD (n=9): 83 ± 31.6 U/L). Normal range for C57/Bl6 (without hepatic tumors) AST: 46–221 U/L (28).

### Nelitolimod delivered via PEDD reduced MDSC, CD4^+^ T cells and increased cytotoxic CD8^+^ T cells within LM

**Figure 5:**
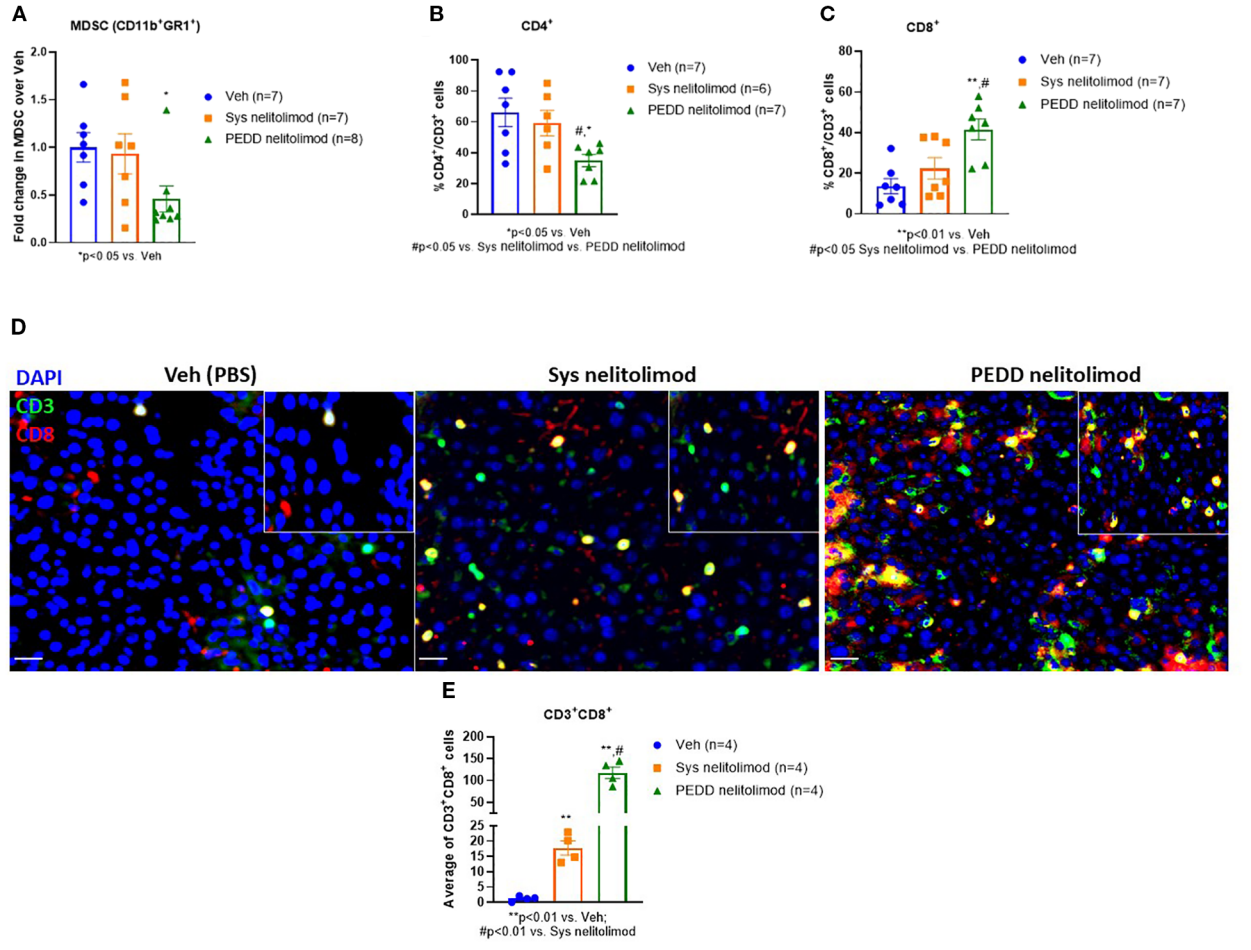
PEDD nelitolimod reduced MDSC, CD4^+^ T cells and increased cytotoxic CD8^+^ T cells within LM. Liver of tumor-bearing mice were harvested 14 days post-treatment. CD45^+^ cells were isolated from non-parenchymal cells (NPCs). **(A)** MDSC cell population (CD11b^+^Gr1^+^), **(B)** CD4^+^ T cells and **(C)** CD8^+^ T cells were quantified by flow cytometry. **(D, E)** Tumors were isolated from each group, OCT-mounted tissues were sectioned, fixed, and stained for CD3 (green), CD8 (red), CD11b (green), and Gr1 (red). Quantification of CD11b^+^Gr1^+^ MDSCs and CD3^+^CD8^+^T cells and from tumors of mice were performed across 5 fields/mouse and n=4 mice were used per group. Scale (20µm). Data were presented as mean ± SEM from and n was mentioned in the individual graph.

We investigated the status of the immunosuppressive MDSC cells and cytotoxic T cells within the tumor by performing flow cytometry. We found that nelitolimod delivered via PEDD reduced the MDSC and CD4**^+^** cells within the liver significantly compared to Veh (**Figures A, B**). In contrast, the percentage of cytotoxic CD8^+^T cells was elevated (**Figure 5C**) within the tumor. We further confirmed the finding by immunofluorescence and found that there was a significant increase in CD8^+^ T cell infiltration (**Figure 5D**) in PEDD nelitolimod as compared to both Veh and Sys Nelitolimod groups (p<0.01). Conversely, there was a decrease in MDSCs (**Figure 5E**) in the PEDD nelitolimod group as compared to Veh (p<0.05). Therefore, nelitolimod delivered via PEDD promoted better anti-tumor immunity compared to Sys nelitolimod.

## Discussion

The method of delivery can have a substantial impact on the distribution of a therapeutic agent within the body. Infusion into the central circulatory system has historically been employed to deliver a wide range of chemo- and bio-therapeutic agents for the treatment of solid tumors. This mode, however, lacks specificity and results in distribution to off-target tissue. Less than 1% of the therapeutic delivered in this manner typically reaches the targeted tissue (29). Coupled with poor delivery efficiency is the risk of systemic toxicity, thus narrowing or eliminating a therapeutic window for many promising drugs.

Many strategies have been developed to improve drug delivery efficiency. One of the easiest, conceptually, is the direct injection of a therapeutic agent into a tumor mass. This methodology has the advantage of delivering highly concentrated therapeutic directly to the target tissue. However, the zone of diffusion is often limited, resulting in heterogeneous distribution of the therapeutic within the target tissue (30). Complex injection needle designs have been developed to reduce this issue, but there are conflicting data regarding meaningful improvement in tissue uptake as a result (31–33). Importantly, TLR9 agonists target immune cells and not tumor cells. As such, intravascular delivery is preferred over needle injection to enable access to liver tumor tissue and peri-tumoral parenchyma where target immune cells may be located. Moreover, therapeutics injected within the liver displayed relatively limited diffusion within the tissue, often being confined to one to two 1 cm thick sections of the liver. This distribution pattern would be insufficient to treat multifocal diffused disease.

PEDD offers an alternative mode of therapeutic delivery. PEDD is achieved using a catheter system that is equipped with a one-way dynamic microvalve structure. When placed within the bloodstream, the valve physically modulates blood flow and pressure. Forward blood flow is retained after device placement, allowing for the migration of therapeutics downstream into the target vascular network. During infusion, pressure can be generated locally within the arterial network without risk of reflux into non-target tissues. This unique hemodynamic modulation enables enhanced penetration of large molecules, such as monoclonal antibodies, into solid tumors. We have previously shown that PEDD significantly increased intra-tumoral levels of anti-PD-1 antibodies in oncopig liver tumors, achieving higher concentrations at the tumor margin compared to conventional infusion (34). This margin is enriched with immune effector cells critical for checkpoint inhibitor activity, underscoring the translational relevance of PEDD as a strategy to optimize antibody-based therapies. PEDD can overcome physical barriers to antibody penetration, enhancing local immune modulation and making it a promising strategy to improve therapeutic outcomes in clinical applications.

In this study, normal porcine liver tissue was treated using a PEDD device with the goal of quantifying distribution and tissue uptake of the label ODN sequences. Using quantitative near-IR imaging of 1cm slices of liver tissue, the full volume of tissue exposed to and retaining the labeled compound was quantified. Unsurprisingly, the volume of tissue exposed to the ODN after PEDD infusion was over 7 times greater than that exposed by needle injection (99.0 ± 28.8 cm^3^ SE vs. 13.5 ± 2.9 cm^3^ respectively). Unexpectedly, the total luminous intensity, a measure of therapy uptake, was significantly greater in PEDD treated tissue as well (48997 ± 10088 lu SE PEDD vs. 19275 ± 6352 lu SE needle injection, 2.5-fold increase).

Direct needle injection was associated with significantly lower luminous intensity, which was observed relative to PEDD. This may be associated with the phenomena known as backflow, which results when the infusate migrates around the needle and flows out from the needle tract (35). Several factors influence the magnitude of this phenomenon, such as needle insertion rate, insertion angle, infusion rate, needle diameter, and tissue pressure (35). Backflow has been implicated in the reduction of efficacy of therapeutic injected into tumors (35). We hypothesize that a substantial portion of the infused ODN volume experienced backflow through the needle tract into the abdomen, resulting in lower tissue retention.

PEDD performed using a microvalve equipped device physically modulates blood flow and local vascular pressure (36), potentially contributing to the high levels of ODN retention observed in the study. At initial placement, the device induced a pressure gradient from 96.8mmHg ± 6.2mmHg SE proximal to the valve to 57.6mmHg ± 8.3mmHg SE distal to the valve (n=8 measurements). Reduction in pressure also results in slower blood flow velocities. During infusion, the therapeutic likely experienced greater contact time with the tissue, resulting in robust absorption.

Such modulation in pressure is also believed to induce redistribution of blood flow within the organ whereby more hypervascular structures, such as tumors, will tend to receive a disproportionate percentage of the flow (36, 37). Pressure modulation can also induce local contraction in normal vessels while tumor tissue, lacking ordered vascular muscular structure, remains patent (38). The net effect of these physiological phenomena is preferential blood flow to tumor tissue and a reduction in flow to normal tissue. This effect has been observed clinically after administration of 99m–MAA. The use of a PEDD device resulted in significantly more MAA deposition in tumor tissue while significantly reducing delivery to normal tissue relative to conventional catheter systems (13).

Furthermore, distal vascular pressure can be temporarily increased due to the added volume of fluid injected during infusion as reflux is prevented by the valve, directing all flow into the target distal vasculature. The temporary increase in pressure during infusion may, in turn, promote penetration of therapeutic into resistive high-pressure regions of the solid tumor tissue. This hypothesis is supported by analysis of the distribution of nelitolimod in and around tumors present in the oncopig model. The use of PEDD with a TriNav device significantly increased the concentration of labeled nelitolimod within and around the periphery of the tumor relative to infusion with a traditional microcatheter. In PERIO-01 Phase I trial (uveal melanoma with liver metastases), serial plasma sampling (pre-infusion, 30 min, 2 hours and 4 hours) demonstrated that PEDD-delivered nelitolimod achieved elevated tumor drug concentration while systemic levels remained transient (17). This effect is not specific to nelitolimod since we have previously demonstrated a similar enhanced delivery of therapeutic microspheres using a similar oncopig model (39, 40). Based on the results of this study, PEDD with a TriNav device increased the overall uptake of the therapeutic in liver tissues and preferentially enriched concentrations in and around the tumors themselves. Also, the biosafety profile of nelitolimod delivered via PEDD was comparable to systemic delivery, as evidenced by the *in vivo* readouts in nelitolimod monotherapy-single dose using a preclinical murine tumor model. Nelitolimod did not affect key metabolic parameters (bilirubin, total protein, albumin, globulin, BUN, ALT, or creatine kinase) irrespective of delivery method, suggesting preserved hepatic and systemic metabolic function. Although AST was transiently elevated on day 3 in both systemic and PEDD groups, levels normalized by day 14 and remained within reported physiological ranges for C57/Bl6 mice without hepatic tumors (28). Overall, these findings suggest that the PEDD-mediated delivery of single dose nelitolimod monotherapy does not introduce any additional metabolic or biosafety risks beyond those already associated with systemic administration. This safety profile, combined with enhanced local delivery, supports the translational feasibility of PEDD as a strategy to optimize nelitolimod activity in solid tumors.

The highly immunosuppressive environment in the LM driven by MDSCs, sinusoidal endothelial cells, dendritic cells, and tolerogenic Kupffer cells acts as a major obstacle in the success of immunotherapy. LM contains an abundance of MDSCs and Tregs, which are critical drivers of the immunosuppressive TME in the liver. MDSC plasticity and programming is organ-specific and can be modified (41), making the regional intravascular infusion of immunotherapeutic agents via PEDD attractive by skewing the MDSC differentiation to promote anti-tumor immunity. We have previously shown in murine LM and pancreatic tumor models that high-pressure PEDD of nelitolimod via the portal vein and pancreatic retrograde venous infusion respectively, in combination with systemic anti-PD-1 demonstrated improved tumor control in both models (6, 26, 42). Here, we have observed a decrease in total liver MDSCs and CD4^+^ T cells with a significant increase in CD8^+^ T cell infiltration. Studies have shown that immunomodulators show a decrease in CD4^+^ T cells and an increase in CD8^+^ T cells, which could be indicative of an anti-tumor response, potentially due to the depletion of regulatory CD4^+^ T cells and the activation or expansion of cytotoxic CD8^+^ T cells (43, 44). We have also shown that nelitolimod delivered via PEDD in combination with a checkpoint inhibitor (anti-PD-1) delivered either subcutaneously or systemically resulted in similar changes in innate and adaptive immune cells within LM, including depletion of liver MDSC and increased M1-like macrophages in the liver, which are supportive of anti-tumor immunity (26). Previous murine studies have shown that intra-tumoral injection of TLR9 agonists enhances the responsiveness of systemic CPIs in treating solid tumors (9). Contrary to this, direct intralesional needle injection of a TLR9 agonist in combination with CPI did not have any clinical benefit (45). In deep visceral tumors such as in liver metastasis, intravascular high-pressure delivery of immunotherapy agents regionally into the tumors using PEDD has the potential to improve the clinical performance of the drugs by appropriate immunomodulation of the TME. This study also highlights that PEDD-based delivery of nelitolimod effectively delivers the drug throughout the liver in the setting of LM, which may be associated with favorable effects on intravascular pressure and flow (46). In clinical trials, nelitolimod delivered via PEDD has shown a tolerable safety profile with or without CPI, and PK data demonstrate high intrahepatic drug levels with low systemic exposure (17, 47, 48).

## Conclusions

The present study was designed to compare the delivery of near-IR labeled ODNs via conventional techniques (needle injection and TMC delivery) with PEDD infusion using a TriNav device. PEDD significantly improved both signal intensity (an index of drug retention) and tissue volume (an index of drug distribution) when compared to needle injection. Furthermore, PEDD infusion resulted in preferential delivery of nelitolimod into and around the tumor relative to conventional delivery with a TMC. Thus, PEDD could be used to enrich the concentration of therapeutic in and around tumors that are typically resistant to therapeutics delivered by blood flow alone.

Once delivered into the high-pressure murine LM microenvironment, nelitolimod demonstrated the ability to: reduce or eliminate MDSC, increase CD8^+^ cell infiltration into the tumor, and produce anti-tumor efficacy.

## Data Availability

The original contributions presented in the study are included in the article/**Supplementary Material**. Further inquiries can be directed to the corresponding author.
