# Supplementary figures and images for: Pressure enabled drug delivery (PEDD) of nelitolimod increased therapeutic delivery, reduced immunosuppression, and improved efficacy in porcine and murine liver tumor models
Source: Front Oncol. 2025 Oct 6;15:1655794. doi: 10.3389/fonc.2025.1655794 (PMC12536300; doi:10.3389/fonc.2025.1655794)

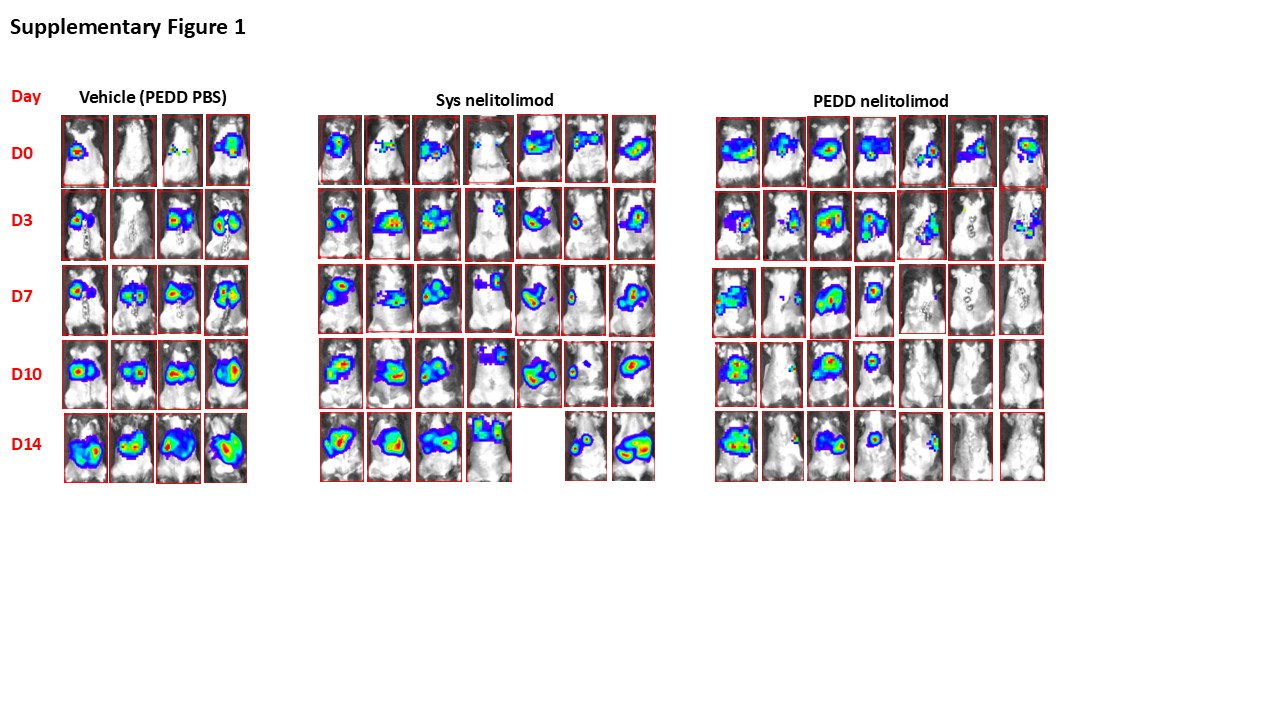

Supplement: Supplementary file 1 [file Image1.jpeg]
